# Ivermectin, a new candidate therapeutic against SARS-CoV-2/COVID-19

**DOI:** 10.1186/s12941-020-00368-w

**Published:** 2020-05-30

**Authors:** Khan Sharun, Kuldeep Dhama, Shailesh Kumar Patel, Mamta Pathak, Ruchi Tiwari, Bhoj Raj Singh, Ranjit Sah, D. Katterine Bonilla-Aldana, Alfonso J. Rodriguez-Morales, Hakan Leblebicioglu

**Affiliations:** 1 Division of Surgery, ICAR-Indian Veterinary Research Institute, Izatnagar, Bareilly, 243 122 Uttar Pradesh India; 2 Division of Pathology, ICAR-Indian Veterinary Research Institute, Izatnagar, Bareilly, 243 122 Uttar Pradesh India; 3 Department of Veterinary Microbiology and Immunology, College of Veterinary Sciences, UP Pandit Deen Dayal Upadhayay Pashu Chikitsa Vigyan Vishwavidyalay Evum Go-Anusandhan Sansthan (DUVASU), Mathura, 281001 India; 4 Division of Epidemiology, ICAR-Indian Veterinary Research Institute, Izatnagar, Bareilly, 243 122 Uttar Pradesh India; 5 Department of Microbiology, Institute of Medicine, Tribhuvan University Teaching Hospital, Kathmandu, Nepal; 6 Semillero de Zoonosis, Grupo de Investigación BIOECOS, Fundación Universitaria Autónoma de las Américas, Sede Pereira, Pereira, Risaralda Colombia; 7 Public Health and Infection Research Group, Faculty of Health Sciences, Universidad Tecnologica de Pereira, Pereira, Colombia; 8 Faculty of Medicine, Grupo de Investigacion Biomedicina, Fundacion Universitaria Autonoma de las Americas, Pereira, Risaralda Colombia; 9 Department of Infectious Diseases, Samsun Liv Hospital, Samsun, Turkey

**Keywords:** COVID-19, 2019-nCoV, SARS-CoV-2, Coronavirus, Therapeutics, Ivermectin

The recent report by Caly et al., describing the antiviral potential of ivermectin against the severe acute respiratory syndrome coronavirus 2 (SARS-CoV-2) in vitro arrive to the agenda of potential candidates for COVID-19 treatment [1]. This discovery gave hope to the researchers who are screening for drugs that can be repurposed for treating the Coronavirus Disease 2019 (COVID-19). Ivermectin, is a member of the avermectin family (Fig. 1); as these compounds are produced by the soil microorganism, *Streptomyces avermitilis,* they are called avermectins [2]. Ivermectin has showed a wide range of activities, ranging from broad-spectrum endo/ecto-parasiticide activity to antiviral, antibacterial, and anticancer activities [3]. It was first introduced commercially in 1981 for use in animals. In addition to being used for treating billions of livestock and companion animals worldwide to help maintain food production and animal health, ivermectin is also used for treating several diseases in humans, e.g. a key drug in the elimination programs of onchocercosis [3, 4]. Ivermectin is considered a drug of choice for various parasitic diseases. As an anthelmintic drug, its mechanism of action in invertebrates mainly involves the opening of glutamate-gated and Gamma aminobutyric acid (GABA)-gated chloride channels, leading to increased conductance of chloride ions and causing subsequent motor paralysis in parasites [5].

**Fig. 1:**
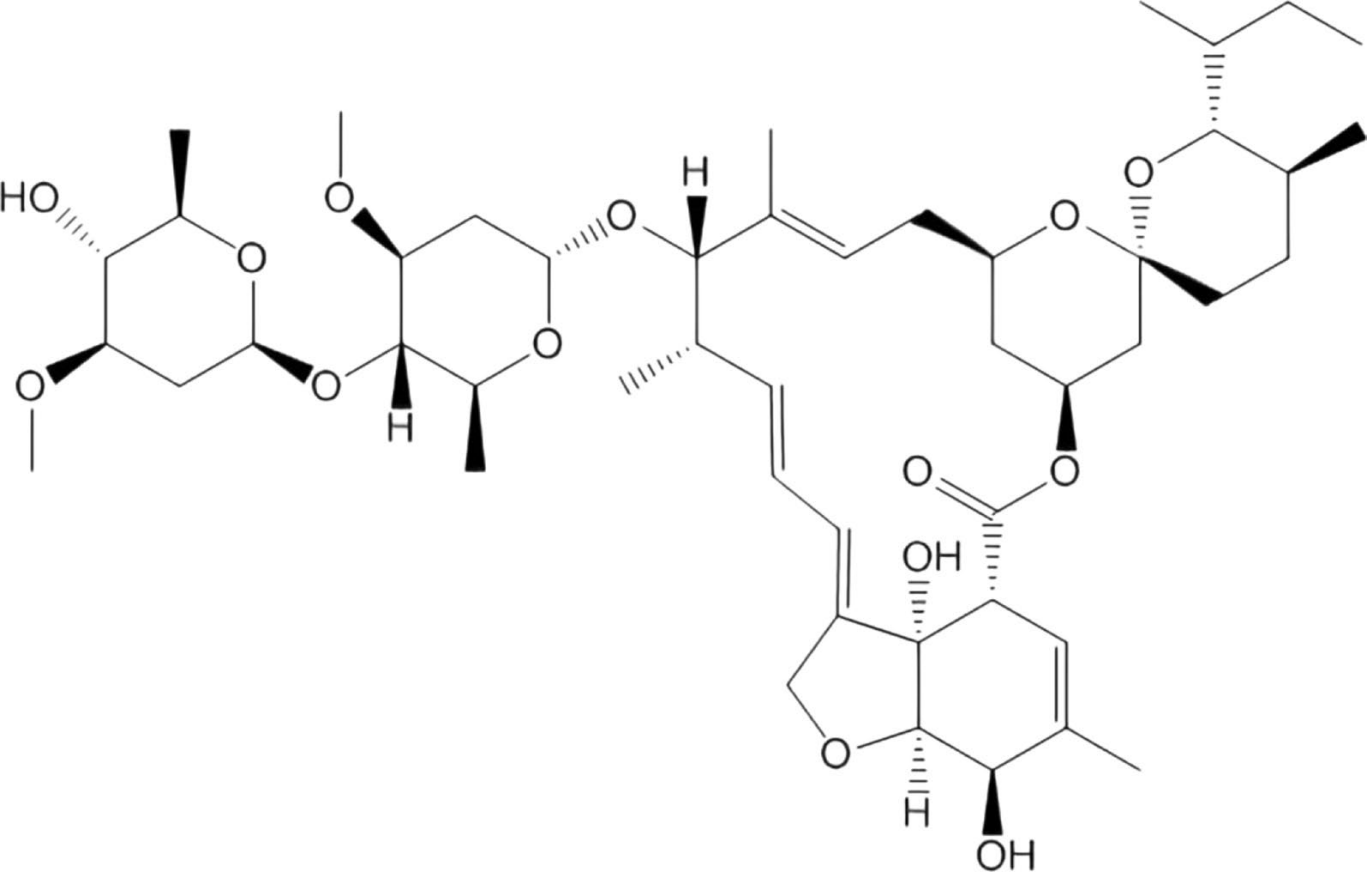
Chemical structure of ivermectin, the 22, 23-dihydro derivative of a macrocyclic lactone avermectin B1

This is not the first time that ivermectin has exhibited antiviral potential against human and animal viruses. The first report on the in vivo effectiveness of ivermectin against viruses demonstrated its effect against parvoviruses in a freshwater crayfish (*Cherax quadricarinatus*) model [6]. This broad-spectrum endo/ecto-parasiticide has exhibited potent antiviral effects against several ribonucleic acid (RNA) viruses, such as Zika virus [7], influenza A virus [8], Venezuelan equine encephalitis virus [9], West Nile virus [10], porcine reproductive and respiratory syndrome virus [11], Newcastle disease virus [12], chikungunya virus [13], human immunodeficiency virus (HIV-1) [14], yellow fever virus, dengue virus, Japanese encephalitis virus, and tick-borne encephalitis virus [15]. However, the in vivo antiviral potential of ivermectin has only been reported against the West Nile virus [10] and Newcastle disease virus [12]. It has been demonstrated that ivermectin showed strong antiviral activity against Newcastle disease virus at a concentration of 100 μg/ml, and exerted cytotoxicity in primary chick fibroblast cells [12]. Ivermectin has also exhibited antiviral activity against deoxyribonucleic acid (DNA) viruses, such as the pseudorabies virus [16], porcine circovirus 2 [17], parvoviruses [6], and bovine herpesvirus 1 [18]. However, the in vivo antiviral potential of ivermectin has only been reported against the pseudorabies virus [16] and parvoviruses [6].

In the study by Caly et al., Vero-hSLAM cells were treated with ivermectin after 2 h of SARS-CoV-2 infection, resulting in ~5000-fold reduction in viral RNA after 48 h [1]. Although the positive result obtained in the in vitro studies suggests the possible in vivo antiviral potential of ivermectin, further validation using an efficient in vivo model is still required. As a matter of concern, we should also consider our previous experience with the in vivo antiviral potential of ivermectin against the Zika virus. Even though its antiviral activity was proven in vitro [7], ivermectin was ineffective at preventing lethal Zika virus (Senegal strain) infection in Ifnar1-knockout mice [19]. Even though ivermectin has exhibited antiviral activity against several RNA viruses in vitro, further studies in in vivo models have been conducted against only a few of these viruses [10, 12].

Ivermectin was previously found to inhibit flavivirus replication by specifically targeting the activity of non-structural 3 helicase (NS3 helicase) in vitro. It is a potent inhibitor of the yellow fever virus and a weak inhibitor of other flaviviruses, such as Japanese encephalitis, dengue, and tick-borne encephalitis viruses [15]. Ivermectin was also found to inhibit the nuclear accumulation of HIV-1 integrase and the non-structural protein 5 (NS5) polymerase of the dengue virus, a phenomenon that is dependent on importin α/β nuclear transport [14]. The broad-spectrum antiviral potential of ivermectin against several RNA viruses is due to its ability to specifically inhibit importin α/β-mediated nuclear transport, which in turn blocks the nuclear trafficking of viral proteins [20]. Several RNA viruses depend on Impα/β1 during the process of infection [21]. SARS-CoV-2, is an RNA virus, is expected to show a similar mechanism of action. The proposed anti-SARS-CoV-2 action of ivermectin involves the binding of ivermectin to the Impα/β1 heterodimer, leading to its destabilization and prevention of Impα/β1binding to the viral proteins. This prevents viral proteins from entering the nucleus, thereby reducing the inhibition of antiviral responses and leading to an efficient antiviral response [1].

The antiviral activity of ivermectin is also found to be related to other mechanisms. Ivermectin has been reported to suppress the replication of the pseudorabies virus by inhibiting the nuclear import of UL42 (an accessory subunit of DNA polymerase) [16]. A similar mechanism of inhibition was reported for another DNA virus, bovine herpesvirus 1 [18]. Ivermectin inhibits the nuclear localization signal-mediated import of capsid protein (Cap) of porcine circovirus 2 [17]. It is, therefore, necessary to identify the exact mechanism underlying the in vitro antiviral activity of ivermectin against SARS-CoV-2 to obtain an insight into the possible mechanism of infection. An overview of the potential modes of the antiviral action of ivermectin is presented in Fig. 2.

**Fig. 2:**
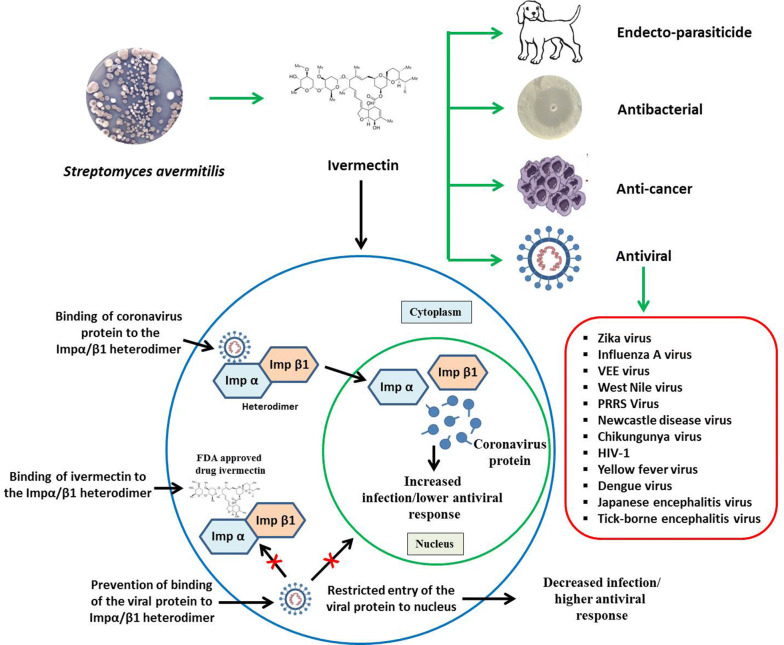
Potential modes of anti-viral actions of ivermectin

It has also been hypothesized that combination therapy using hydroxychloroquine and ivermectin may exert a synergistic inhibitory effect on SARS-CoV-2. In this combination, hydroxychloroquine acts by inhibiting the entry of SARS-CoV-2 into the host cells, whereas ivermectin further enhances the antiviral activity by inhibiting viral replication [22]. Considering the promising result of the in vitro study, the clinical benefit of ivermectin therapy was evaluated in an observational registry-based study involving critically ill SARS-CoV-2-infected patients. Treatment with ivermectin at a dose of 150 μg/kg was found to be associated with a lower mortality rate and reduced healthcare resource use [23]. Even though the result of this preliminary study provides hope for the utilization of ivermectin in a clinical setting, further evaluation in randomized clinical control trials is required before this wonder drug can be adapted into treatment guidelines, as has been occurring with other drugs under use and investigation in COVID-19, such as chloroquine [24].

Besides, although ivermectin has been reported to exert potent antiviral activity against many viruses, its application is mainly hampered by pharmacokinetic problems such as high cytotoxicity and low solubility. To overcome these problems, various liposomal systems have been engineered and used as ivermectin nanocarriers in several cell lines, which resulted in lower cytotoxicity than that of free ivermectin [25]. Before considering ivermectin for widespread use as an antiviral agent, detailed in vivo and in vitro investigations of its effect in various animal models and cell culture systems are of utmost importance.

The in vitro antiviral activity of ivermectin against SARS-CoV-2 has further extended the antiviral spectrum of this drug. As ivermectin is an United States Food and Drug Administration (FDA)-approved drug, repurposing it for anti-SARS-CoV-2 therapy will not be a problem. Nevertheless, the real question is, will it reach the stage of randomized clinical control trials in SARS-CoV-2-infected patients, or will it fail in the in vivo study stage? Although no clinical trials have reported its efficacy and safety in the context of COVID-19 yet, is expected to see in the near future them, delivering information about its potential therapeutic action in the clinical setting.

Hence, we can conclude the following:

- Ivermectin exerts broad-spectrum antiviral activity against several animal and human viruses, including both RNA and DNA viruses.
- The antiviral potential of ivermectin against various viruses is mediated via the targeting of the following: importin α/β-mediated nuclear transport of HIV-1 integrase and NS5 polymerase; NS3 helicase; nuclear import of UL42; and nuclear localization signal-mediated nuclear import of Cap.
- As SARS-CoV-2 is an RNA virus, the antiviral activity of ivermectin may be mediated through the inhibition of importin α/β-mediated nuclear transport of viral proteins.
- The clinical efficacy and utility of ivermectin in SARS-CoV-2-infected patients are unpredictable at this stage, as we are dealing with a completely novel virus.

## Data Availability

If required.
